# Mixed phase iron oxides thin layers by atmospheric-pressure chemical vapor deposition method

**DOI:** 10.1016/j.mex.2024.102940

**Published:** 2024-09-04

**Authors:** Vladimir Parra-Elizondo, Ana Karina Cuentas-Gallegos, Daniella Pacheco-Catalán

**Affiliations:** a Unidad de Energía Renovable, Centro de Investigación Científica de Yucatán, A.C, Sierra Papacal 5Km, CP. 97200, Mérida, Yucatán, México; b Centro de Nanociencias y Nanotecnología, Universidad Nacional Autónoma de México, Ensenada, B.C. México

**Keywords:** Atmospheric pressure CVD, Mixed-phase iron oxide, Thin layer oxide, Mixed phase iron oxides thin layers by Atmospheric-Pressure CVD method

## Abstract

This paper provides a simple method for producing a metal oxide thin layer methodology by atmospheric pressure chemical vapor deposition (APCVD) synthesis over stainless steel substrates. This methodology enables the formation of thin iron oxide layers at its performance at various temperatures of 330 °C, 430 °C, and 530 °C. The deposition arises from thermal decomposition of the iron organometallic precursor Fe_3_(CO)_12_, forming a thin layer of iron oxide is, by the ozone present in the reaction chamber promoting the deposition of the iron oxide particles over the substrate. The Raman characterization suggest that at 330 °C, a mixture of hematite and magnetite is predominant on the as deposited substrates, also hematite modes show to be more pronounced as the band at 300 cm^-1^ narrows. Conversely, magnetite is prominent at higher synthesis temperatures, exhibiting a more intense Eg5 mode at 680 cm^-1^. The particles exhibit a uniform morphology, with both metal oxide phases coexisting. The average diameter of the particles is 50 nanometers as scanning electronic microscopy shows in a transversal sample section.

• The formation of particles is attributed to the combination of iron ions ^+^2 and ^+^3 in the deposition process and their interaction with oxygen in the given synthesis parameters at atmospheric pressure chemical vapor deposition (APCVD).

## Graphical abstract

**Figure:**
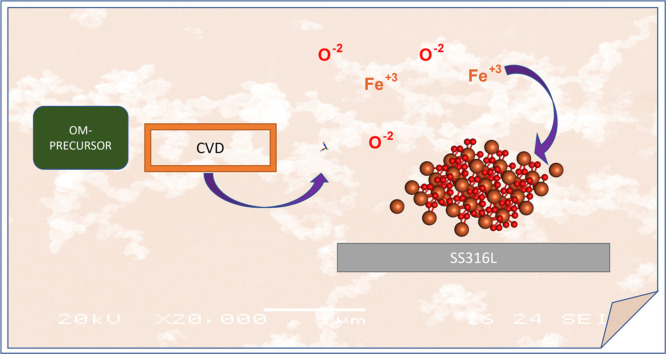

Specifications table

**Table:** 

Subject area:	Materials Science
More specific subject area:	Synthesis and characterization of nanostructured materials, thin layer oxides
Name of your method:	Mixed phase iron oxides thin layers by Atmospheric-Pressure CVD method
Name and reference of original method:	Not applicable
Resource availability:	Not applicable

## Background

The Chemical Vapor Deposition (CVD) is a technique employed to fabricate thin films and nanostructures [[1], [2], [3]]. Its significance lies in its ability to produce materials with tuned properties such as thickness, surface active materials with electrical activity, a crucial factor in the development of high-performance devices such as photocatalysis [4,5], hydrogen production [6], adsorption processes, water treatment, and energy storage materials [[1], [2], [3],^6^]. The process involves the deposition of gaseous reactants onto a substrate, where they undergo reaction or decomposition to form solid materials. CVD's capacity for precise thickness control, uniformity over large areas and complex geometries, composition tunability, and high purity output make it suitable for various industries. These include semiconductor manufacturing, solar cell production, optical coatings, and protective layer applications. The technique's adaptability in creating structures ranging from simple thin films to complex nanoarchitectures further underscores its importance in advancing technological capabilities across multiple fields.

The process of producing thin-layer materials using CVD-manufactured materials does not involve a straightforward setup that enables rapid production, unlike other methods that assemble comparable nanostructures through multiple stages such as electrochemical deposition [7], laser-induced plasma [5], which require additional reagents, pH control agents, and extended synthesis durations [6,7]. Hence, this methodology minimizes expenditures associated with infrastructure investment, energy utilization, and surplus reagents.

The synthesis methodology entails the thermal degradation of organometallic precursor in a segregated chamber to generate a vapor fraction. This synthesis begins with the precursor vapor, which is subsequently conveyed to the interior of a tubular reactor containing a chosen substrate placed within a temperature-regulated chamber utilizing an inert carrier gas. The reactor's high temperature facilitated the decomposition of the precursor vapor fraction and the formation of the desired material in the presence of an oxidation agent. The resulting gases were then collected in a water trap, where the excess was removed and separated in a fume filter. The temperature of the furnace is a critical factor that provides the necessary thermal energy for the synthesis process and is utilized to investigate the phase formation of the thin film deposits. The temperature range varied between 330° and 530 °C, in order to investigate if there was an effect between temperature and phases

## Value of the protocol

• A straightforward approach to assembly, without complex apparatus or vacuum equipment.
• Thin-layer oxides can be generated in a single, simple step without the need for vacuum equipment, additives, or templates.

## Method details

### Description of the experimental components apcv system

**Fig. 1:**
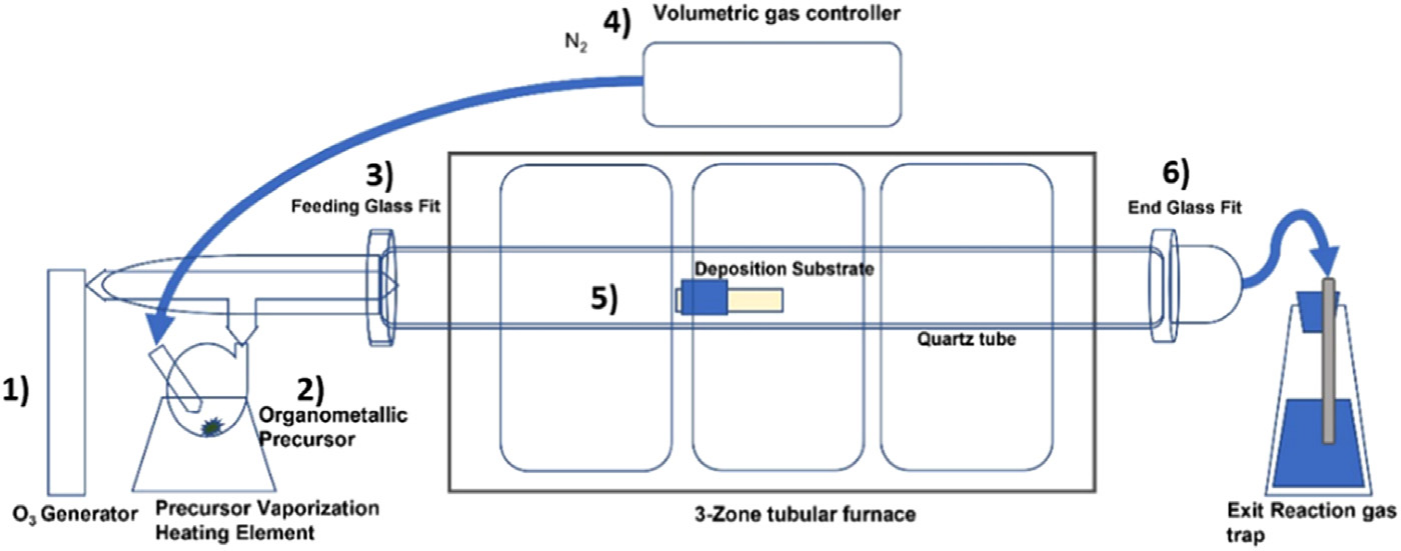
Diagram of system elements with the representations of main components.

**Fig. 2:**
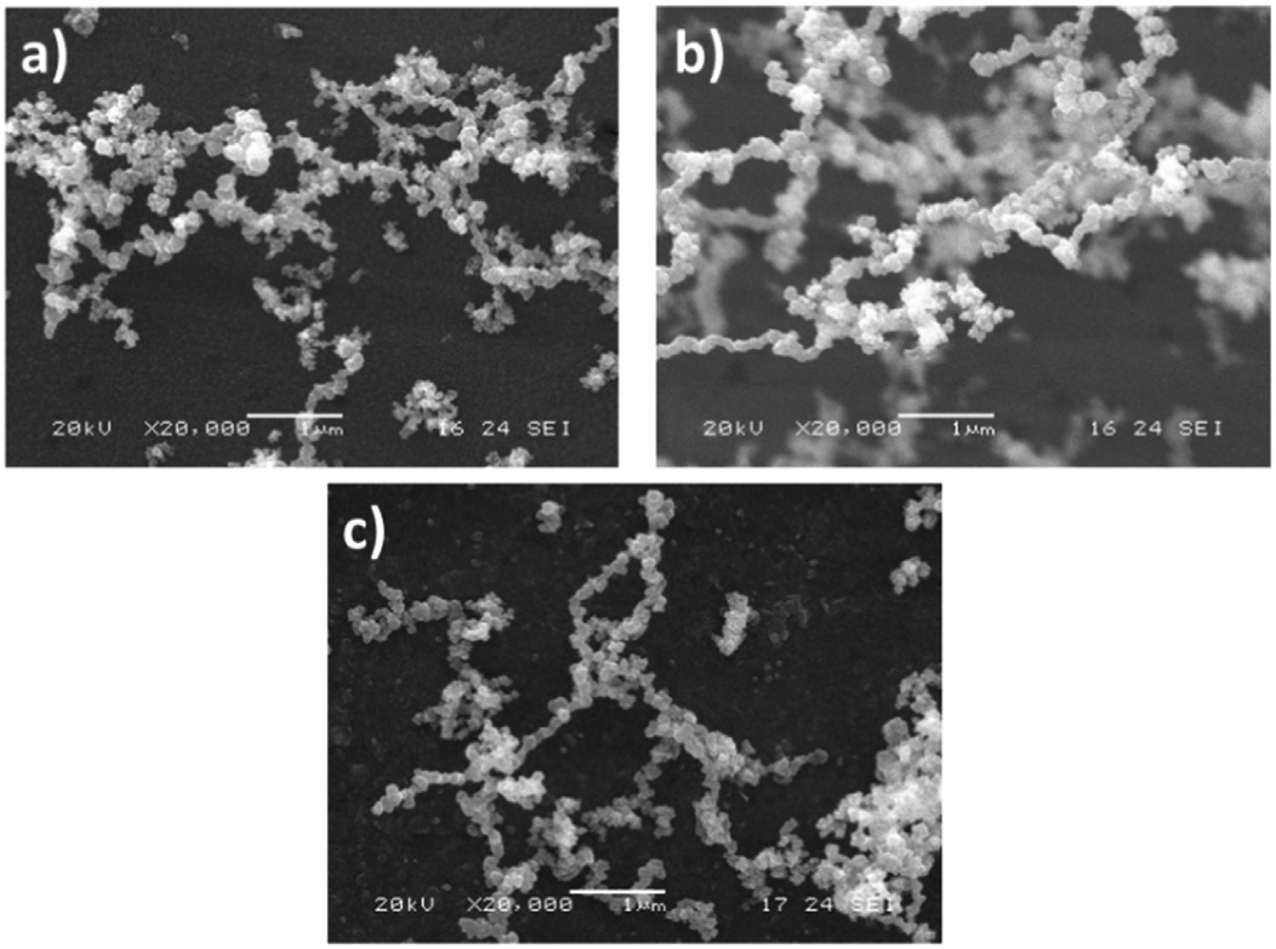
SEM of the oxide films obtained at a) 330, b) 430, and c) 530 °C.

In Figs. 1 and 2a schematic representation of the system used which consists of the following parts:

(1) Ozone generator
(2) Precursor thermal vaporizer
(3) Feeding gas fit
(4) Volumetric gas controller
(5) Reaction chamber
(6) Gas exit

## Materials and equipment required

For the synthesis of the thin films of mixed iron oxide, it is used as the substrate of 20 × 10 × 0.5 mm of stainless steel 316 L. Triiron dodecaarbonyl was used as precursor; as carrier gas and catalyst gases were used, nitrogen and ozone were used, respectively. The reaction chamber was a quartz tube with dimension of 1 m x 0.0254 m of length and diameter, respectively. The tube was held by a refractory cement holder to place the substrate in the reaction chamber in the tubular furnace (Tables 1 and 2).

**Table 1:** Specifications of the reagents.

Reagent	Chemical formula	Molecular weight	supplier	CAS
Nitrogen gas	N_2_	28.02	Praxair	7727–37–9
Ozone	O_3_	48	N/A	N/A
Triiron dodecacarbonyl	Fe_3_(CO)_12_	503.66	Sigma Aldrich	17,685–52–8

**Table 2:** Description of the equipment.

Equipment	Trademark	Model
Ozone generator	Mountainlife	ML-1.5 g21.
Tubular Furnace	Thermo Scientific Lindberg Blue M	STF55346C-1
Volumetric gas controller	Electrochem, Inc.	MTS-A-150
Heating Mantle	United	UNEHMTLE-250

## Experimental procedure

The following steps occur sequentially and comprehend the experimental APCVD system:

(a) The addition of the precursor (100 mg) into the modified round heating baker. Connection of the line for the carrier gas from the volumetric gas controller to the modified round precursor heating gas port. Assure sealing between fittings in the pair feeding glass fixture.
(b) Undertake the connection of the O_3_ generator outlet gas tube to the feeding glass manifold such that it accommodates the second gas inlet in the feeding glass fixture.
(c) Place the deposition 316 L substrate over the refractory cement holder, positioned within the quartz tube at the half of tube length.
(d) Securely fasten both the feeding glass fittings and the end glass fittings on opposite sides of the quartz tube and establish a gas exhaust connection to the water trap at the quartz tube's terminal end.
(e) Undertake the system's purge by employing a constant flow of N_2_ at 60 sccm for 10 min. This procedure also aims to verify the efficacy of the sealing intersections and identify any potential gas leaks within the synthesis system.
(f) Direct the heating element for precursor vaporization to 160 °C to sublime the organometallic precursor.
(g) Undertake the initiation of the tubular furnace heating program with a temperature increase at a rate of 10 °C per minute, until the designated experimental temperature of 330 °C, 430 °C, or 530 °C is maintained, subsequently holding the temperature for 60 min.
(h) Monitor the temperature of the heating element for the organometallic precursor with a thermometer. When the temperature has reached 160 °C, activate the N_2_ displacement gas at a flow rate of 60 sccm to initiate the deposition process.
(i) Initiate the O_3_ generator and stabilize the outlet flow rate at 60 sccm for an hour.
(j) Upon the reaction time is completed (60 min), shut down the O_3_ generator and carefully remove the quartz tube from the system when the temperature of the system is at room level.
(k) Extract the as-deposited substrates for characterization.

## Method validation

The purpose of this study was to investigate the effect of deposition temperature and iron oxide formation ranging from 330 °C to 530 °C. The difference in temperatures promoted mixed-phase deposition. The formation of the thin films was optimal within this temperature range. The morphology from the as-deposited samples was analyzed by SEM. The films exhibit a cubic apparent shape with an irregular surface morphology independent of the synthesis temperature [[8], [9], [10], [11]].

**Fig. 3:**
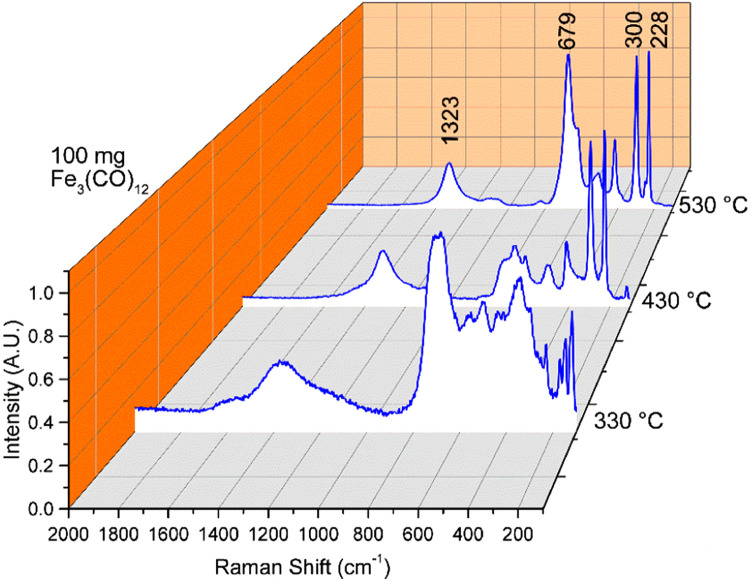
Raman spectra of the e oxide films obtained at a) 330, b) 430, and c) 530 °C.

The Raman characterization of thin oxide films is illustrated in Fig. 3. The variations observed may be attributed to the changes and intensities of responding modes. The Raman modes identified in the characterization can be assigned as follows: 1323 cm^-1^, which could indicate the symmetric stretching mode of some Fe-O bonds. The Raman shift around 679 cm^-1^ is assigned to the Fe-O bending modes. The vibration between iron and oxygen atoms can also be found at around 300 cm^-1^ [12]. Lastly, 228 cm^-1^ is associated with the translational modes or lattice vibrations [12]. Also, peaks located at 225, 245, 292, 411,498, 611 and 1323 cm^−1^ correspond to α-Fe_2_O_3_. A small amount of Fe_3_O_4_ can also be detected as a peak at 663 cm^−1^ [12].

The method was validated using the characterization techniques mentioned in the section. It successfully produces oxide thin films with the proposed characteristics and chemical mixed composition, which may find applications in electrochemical storage systems [[9], [10], [11]]*.*

## Limitations

The experiments were carried out atmospheric pressure at sea level.

## CRediT author statement

The authors contributed to the present study as follows. **Vladimir Parra-Elizondo:** Conceptualization, Methodology, Investigation, Writing-original draft. **Ana Karina Cuentas Gallegos:** Investigation, Resources, Project administration. **Daniella Esperanza Pacheco-Catalan:** Writing-review & editing, Methodology, Supervision, Investigation, Resources.

## Declaration of competing interest

The authors declare that they have no known competing financial interests or personal relationships that could have appeared to influence the work reported in this paper.

## Data Availability

Data will be made available on request.
